# Reviewed article: Oliveira GG, Gonçalves AS, Wermann H, Artus RG, Groppelli F, Gonçalves NNS. Trend in the detection rate of syphilis among older adults: time series analysis, Brazil, 2014-2023 Epidemiol Serv Saude. 2026:35;e20250900.

**DOI:** 10.1590/S2237-96222026v35e20250900.a

**Published:** 2026-04-10

**Authors:** João Paulo Cola

**Affiliations:** 1 Prefeitura Municipal de São Mateus, São Mateus, ES, Brazil

## Peer review A

## Questionnaire

Does the manuscript contain new and significant information to justify publication? Yes

Does the Abstract (Summary) clearly and accurately describe the content of the article? No

Is the problem significant and concisely stated? Yes

Are the methods described comprehensively? Yes

Are the interpretations and conclusions justified by the results? Yes

Is adequate reference made to other work in the field? Yes

## Manuscript Structure

Length of article is: Adequate

Number of tables is: Too few

Number of figures is: Too few

Please state any conflict(s) of interest that you have in relation to the review of this paper. None

Interest: Good

Quality: Good

Originality: Average

Overall: Good

Recommendation: Major Revision

## Comments

Comentários críticos:

1. O objetivo da pesquisa está bem definido e delineamento escolhido é adequado. A escolha do modelo de regressão por pontos de inflexão (Joinpoint regression) para análise da tendência temporal das taxas de detecção de sífilis e adequado para este tipo de estudo. Contudo, alguns aspectos da descrição da metodologia merecem maior detalhamento, a fim de garantir maior clareza, transparência e robustez analítica. Foi estabelecido previamente um único ponto de inflexão em 2020, sem uma justificativa plausível declarada na metodologia (COVID-19 ?). Seria importante que os autores explicitassem os critérios adotados para essa decisão. Além disso, questiona-se se foi considerada a possibilidade de existência de outros pontos de inflexão ao longo da série, e como a fixação exclusiva em 2020 pode ter influenciado os resultados.

2. O intervalo pós-2020 abrange apenas três anos, o que pode comprometer a estabilidade das estimativas e aumentar a influência de flutuações anuais. Seria interessante que os autores comentassem como interpretam esse segmento mais curto e suas implicações na robustez dos achados. O intervalo curto de análise pode ter limitado a análise e direcionado para uma estabilidade da série temporal.

3. O texto não descreve se houve avaliação de pressupostos fundamentais do Joinpoint regression, como independência dos resíduos, homocedasticidade e ausência de autocorrelação temporal. Solicito que os autores esclareçam se tais verificações foram realizadas e, em caso afirmativo, quais procedimentos foram adotados.

4. Séries de sífilis podem ser particularmente sensíveis a oscilações decorrentes de subnotificação, surtos localizados ou alterações em sistemas de vigilância. Pergunta-se se os autores realizaram alguma análise de sensibilidade ou inspeção dos dados para identificar outliers que pudessem impactar a posição ou o número de pontos de inflexão.

5. O modelo, por ser linear em segmentos, não captura possíveis padrões não lineares ou variações sazonais. Seria relevante que os autores discutissem essa limitação, bem como a justificativa para não considerar outras abordagens comparativas (por exemplo, modelos ARIMA ou GAMs).

6. Avaliar a inclusão de um gráfico que represente a distribuição temporal da taxa de detecção de sífilis em idosos ao longo do período analisado, o que possibilitaria melhor visualização das tendências.

7. O resumo deve ser revisado de modo a apresentar com maior clareza os principais resultados, devidamente sustentados pelos métodos empregados. Ressalta-se a importância de explicitar, de forma objetiva, como foi avaliada a tendência da série temporal (crescente, decrescente ou estável), de modo a subsidiar a adequada interpretação dos achados. Da mesma forma, os resultados devem ser reavaliados, de modo que as conclusões apresentadas estejam apoiadas em dados numéricos, incluindo os valores de AAPC encontrados, garantindo consistência entre métodos, resultados e interpretação.

Comentários importantes:

1. O manuscrito apresenta resultados relevantes, contudo, a seção de discussão necessita ser aprofundada. Recomenda-se ampliar a reflexão sobre como os achados podem contribuir para o aprimoramento dos serviços de saúde, considerando especialmente o impacto do aumento da sífilis na população idosa e as possíveis repercussões para outras infecções sexualmente transmissíveis. Além disso, sugere-se discutir de forma mais detalhada quais ações podem ser implementadas para modificar esse cenário, apresentando perspectivas concretas dos serviços de saúde voltadas à redução da sífilis nessa população. Esse aprofundamento fortalecerá a aplicabilidade dos resultados e destacará a importância do estudo para a formulação de estratégias em saúde pública.

2. Os resultados são completos e conclusivos. No entanto, na análise descritiva dos casos, todos os casos notificados foram incluídos, o que pode gerar confusão quanto à população efetivamente analisada. Recomenda-se que essa informação seja explicitada na seção de metodologia, detalhando a população analisada, os procedimentos adotados para a análise descritiva, as variáveis utilizadas, as medidas calculadas e a forma de apresentação dos dados.

3. A metodologia apresentada está organizada e coerente com o desenho de estudo. Contudo, para assegurar a replicabilidade da pesquisa e oferecer maior detalhamento metodológico, recomenda-se que o manuscrito apresente de forma mais clara o processo de vigilância da sífilis no Brasil, contemplando a definição de caso conforme o Guia de Vigilância em Saúde do Ministério da Saúde, documento oficial que orienta a notificação e confirmação dos casos no país. Observa-se que a definição descrita no manuscrito diverge daquela estabelecida oficialmente pelo Ministério da Saúde, sendo importante adequar o texto para garantir a conformidade metodológica e a correta interpretação dos resultados.

4. Rever a descrição da população do estudo. Deixar claro quais casos foram incluídos e excluídos para o cálculo da taxa de detecção.

5. A estratégia adotada para estimar as populações anuais de 2014 a 2023 e calcular as taxas de detecção de sífilis demonstra a preocupação em minimizar vieses de aferição associados a denominadores populacionais imprecisos, especialmente frente às divergências identificadas entre as estimativas intercensitárias.alguns pontos metodológicos merecem maior esclarecimento. O uso da taxa de crescimento geométrico anual médio assume um padrão de crescimento homogêneo da população no período 2010-2022. Como variações decorrentes de migração ou mudanças socioeconômicas podem afetar esse padrão, sugiro que os autores justifiquem mais detalhadamente essa escolha e comentem por que não consideraram as estimativas intercensitárias oficiais do IBGE como alternativa ou complemento.

6. A projeção do denominador populacional para 2023, baseada em uma tendência constante de 12 anos, pode ser sensível a eventos recentes (como a pandemia de COVID-19). Pergunta-se aos autores qual o impacto potencial dessa extrapolação nas estimativas de taxa de detecção de 2023 e se foi realizada alguma análise de sensibilidade.

7. No trecho final, a taxa é descrita como sendo calculada pela divisão dos casos de sífilis pelo número de “idosos”, embora a projeção populacional apresentada se refira à população geral. Solicito esclarecimento sobre qual população foi de fato utilizada para projeção.

8. Seria relevante que os autores discutissem em que medida as taxas de detecção poderiam variar caso fossem utilizadas as projeções oficiais do IBGE, de modo a situar o leitor sobre a robustez dos achados frente a diferentes estratégias de cálculo populacional.

9. Há menção a multiplicação por “100.00” na taxa de detecção, o que possivelmente corresponde a 100.000 habitantes. Recomendo corrigir para evitar ambiguidades no cálculo.

Comentários desejáveis:

1. O texto necessita de revisão para garantir maior clareza, precisão e objetividade. Observam-se frases longas e prolixas, que podem comprometer a compreensão. Recomenda-se utilizar linguagem mais ativa e direta, além de reduzir o tamanho dos parágrafos para proporcionar maior fluidez e facilitar a leitura.

2. As tabelas e figuras apresentadas necessitam de revisão a fim de assegurar maior clareza e precisão na comunicação dos resultados. As ilustrações devem ser autoexplicativas, com títulos objetivos e claros quanto ao conteúdo apresentado. Por exemplo: “Taxa de detecção, por 100 mil habitantes, de sífilis em pessoas idosas, segundo faixa etária e região geográfica no Brasil, 2014-2023”.

3. Assegure-se de que as ilustrações contribuam para a clareza e o impacto visual do trabalho. Observa-se que a Tabela 1 e o gráfico correspondente são redundantes, não acrescentando informações complementares.

